# Glandular quinoline-derivates protect crustacean woodlice from spider predation

**DOI:** 10.1098/rsif.2025.0260

**Published:** 2025-08-06

**Authors:** Andreas Fischer, Regine Gries, Camila A. Roman-Torres, Anand Devireddy, Gerhard Gries

**Affiliations:** ^1^ Department of Biological Sciences, Simon Fraser University, Burnaby, British Columbia, Canada; ^2^ Department of General and Systematic Zoology, University of Greifswald, Greifswald, Germany

**Keywords:** antipredator trait, Oniscidia, chemical ecology, spider

## Abstract

In evolutionary time, aquatic crustaceans colonized land and faced new terrestrial predators such as spiders and ants. We tested the hypothesis that the crustacean terrestrial woodlouse *Porcellio scaber* produces defensive metabolites that provide protection against terrestrial predators. When attacked by a predator, *P. scaber* expels proteinaceous secretions from its tegumental glands. Analyses of gland secretion extracts by gas chromatography–mass spectrometry and by liquid chromatography–mass spectrometry revealed four metabolites: methyl 8-hydroxy-quinoline-2-carboxylate, methyl 8-hydroxy-4-methoxy-quinoline-2-carboxylate, methyl 8-(sulfooxy)quinoline-2-carboxylate and methyl 4-methoxy-8-(sulfooxy)quinoline-2-carboxylate, the latter three being natural products not previously known. In behavioural experiments, *Steatoda grossa* spiders readily preyed on *Tenebrio molitor* beetles but avoided chemically well-defended *P. scaber*. When beetles were rendered chemically well-defended by topical applications of either *P. scaber* gland secretion extract or synthetic metabolites identified in these extracts, spiders rejected the beetles as prey. Our data support the hypothesis that *P. scaber* produces defensive metabolites against terrestrial predators. We show that the crustacean *P. scaber*, like many insects, is chemically defended against predators.

## 1. Introduction

In the struggle for survival, predation has prompted the evolution of anti-predator defences in prey [1]. Behavioural adaptations, such as fleeing or feigning death in response to a predator, are widespread across the animal kingdom [2]. Similarly, protective morphological traits, such as hard exoskeletons or spines, have evolved independently in various lineages throughout the tree of life [3,4]. Chemical defences by prospective prey entail the secretion of predator-repelling (foul-smelling) or predator-deterring (foul-tasting) metabolites [5].

Animals often use behavioural, morphological and/or chemical anti-predator defence traits to enhance their survival [5–8]. Chemical defences evolved across many animal taxa, from protozoans to vertebrates, including all life stages [5]. Defensive metabolites serve either in a pre-emptive or a reactive line of defence. Poisonous metabolites on salamander skin exemplify a pre-emptive chemical defence [9], whereas the foul-smelling secretions of a skunk threatened by a predator represent a reactive chemical defence [10]. The bioactive metabolites in these defences are diverse, ranging from complex enzymes in venoms [11] to simple formic acid in defensive sprays of ants [6]. Mostly insect-produced defence metabolites have been identified to date, while not a single defence metabolite produced by crustaceans is currently known [5,12]. This is astounding because crustaceans are close phylogenetic relatives of insects and are taxonomically diverse [13].

Crustaceans are mostly aquatic but polyphyletic ‘Oniscidea’ isopods (woodlice) have colonized land [14,15]. In their adopted terrestrial habitats, early Oniscidea isopods faced new terrestrial predators, prompting the evolution of adaptive defence tactics [16]. These tactics entail behaviours such as grouping with lower predation risk for individuals [17], scattering and fleeing, rolling into a ball (conglobating), or producing stridulatory sound when disturbed [16]. Besides behavioural adaptations, isopods have evolved an array of morphological defences, including a hardened exoskeleton, spiny tergites and morphs with aposematic coloration [16]. The key chemical defence of woodlice pertains to proteinaceous tegumental gland secretions [18] that coagulate within seconds of release and render the woodlice deterrent to potential predators [5,16,18,19], such as ants and spiders [19,20]. The common woodlouse *Porcellio scaber* (Oniscidea: Porcellionidae) is particularly deterrent to predators [18,19].

We tested the hypothesis that *P. scaber,* as a representative terrestrial crustacean, is chemically defended against terrestrial predators. We tested this hypothesis by identifying metabolites in tegumental gland secretions from *P. scaber* and by testing their behavioural effect on a predatory spider. As a generalist predatory spider, we used female false widows, *Steatoda grossa* (Araneae: Theridiidae), which may prey on *P. scaber* [21]. As ‘control prey’, we used *Tenebrio molitor* beetles which are reported to be chemically defended [22,23] but in our pre-screening tests were readily preyed upon by *S. grossa*. We predicted that (i) *S. grossa* preys more readily on *T. molitor* than on chemically well-defended *P. scaber*, and (ii) *S. grossa* rejects *T. molitor* rendered chemically well-defended by treatment with synthetic *P. scaber* secretion metabolites.

## 2. Methods

### 2.1. Animal husbandry

*Porcellio scaber* woodlice were collected near Tsawwassen, British Columbia, Canada (49°0′53.856″ N, 123°2′26.6532″ W) and housed communally in a plastic bin (61 × 39.5 × 21 cm) fitted with mesh-covered holes for ventilation and filled partially with moist C-I-L^®^ Black Earth Top Soil (Riviere-du-Loup, QC, Canada). Woodlice were provisioned with organic potatoes and fish food (Nutrafin basix Staple Food, Rolf C. Hagen Inc., Montreal, Canada) ad libitum*.* Adult *T. molitor* mealworm beetles were housed communally in a similar bin filled with wheat bran and provisioned with organic potatoes and fish food ad libitum [24]. Adult female *S. grossa* spiders were housed singly in translucent 300 ml plastic cups (Western Family, Canada) and fed four *Phormia regina* black blow flies per week [25]. All animals were kept at 22°C under a reversed light cycle (12 : 12 h).

### 2.2. Collection of secretions from *P. scaber* tegumental glands

Defensive secretions from adult *P. scaber* were collected by immobilizing specimens with modelling clay (Craftsmart^®^, Michaels Stores Inc., Irving, TX, USA) and bringing a heated metal probe briefly into contact with the woodlouse’s tergites [18], thus stimulating the release of defensive secretions from their tegumental glands (electronic supplementary material, video S1). Secretions (less than 1 µl per woodlouse) were collected in 5 µl microcapillaries (Drummond Microcaps, Drummond Scientific Comp., Broomall, PA, USA). The secretions from 83 *P*. *scaber* were pooled, dissolved in high-performance liquid chromatography (HPLC)-grade methylene chloride (40 µl per secretion), and then concentrated under a gentle stream of nitrogen to achieve a final concentration of one secretion equivalent per 1 µl. Adult *P. scaber* replaced their compromised integument through moulting.

### 2.3. Chemical analyses of *P. scaber* tegumental gland secretions

In search of defensive metabolites, aliquots (2 µl) of secretion extract were analysed using a 5977A mass selective detector coupled to a 7890B gas chromatograph (GC) (Agilent, Santa Clara, CA, USA) fitted with a DB-5 column (30 m × 0.25 mm ID, film thickness 0.25 µm). The injector port was set to 250°C, the mass spectrometry (MS) source to 230°C, and the MS quadrupole to 150°C. Helium was used as a carrier gas at a flow rate of 35 cm s^−1^, with the following temperature programme: 40°C held for 5 min, 10°C min^−1^ to 280°C (held for 20 min) [26].

In search of very polar metabolites that would not be detectable by gas chromatography–mass spectrometry (GC-MS) analyses [27,28], secretion extract was further analysed by liquid chromatography coupled with mass spectrometry (LC MS). After the extract was concentrated to near-dryness and then reconstituted with acetonitrile, 2 µl aliquots were analysed by LC MS. The system consisted of an Agilent 1200 LC fitted with a Spursil C_18_ column (30 × 3.0 mm, 3 µm; Dikma Technologies, Foothills Ranch, CA, USA) and a Bruker maXis Impact Ultra-High Resolution TOF (UHR-Qq-TOF) mass spectrometer. Electrospray ionization was set to positive (+ESI) at a gas temperature of 200°C and a flow of 9 l min^−1^. The nebulizer was set to 4 bar and the capillary voltage to 4200 V. The column was eluted with a 0.4 ml min^−1^ flow of a solvent gradient starting with 95% water and 5% acetonitrile, and ending with 100% acetonitrile after 4 min [29]. Identified metabolites were synthesized as outlined in the electronic supplementary material.

### 2.4. Testing for defensive functions of *P. scaber* tegumental gland secretions and synthetic gland metabolites

Prediction 1*—S. grossa* preys more readily on *T. molitor* than on chemically well-defended *P. scaber*—was tested in experiments 1–4. In each experimental replicate, a spider, food-deprived for four weeks [30] to enhance her predatory responsiveness, was transferred to an inverted 300 ml cup and offered as prey a *T. molitor* beetle (Exp. 1, *n* = 20) or a *P. scaber* woodlouse (Exp. 2, *n* = 20). Similarly, in experiments 3 and 4 (*n* = 20 each), the spider was offered as prey a beetle treated either with *P. scaber* defensive secretion (1 equivalent) and water (50 µl) (Exp. 3) or with water only (50 µl) (Exp. 4). After 24 h, all replicates were terminated, and predation events were recorded. A dead prey covered in spider silk was deemed preyed upon, whereas a live prey without silk cover was deemed not preyed upon by the spider.

Prediction 2—spiders reject beetles rendered chemically well-defended by treatment with synthetic *P. scaber* secretion metabolites—was tested in experiment 5. With evidence that the synthetic blend (40 ng total) of four *P. scaber* secretion metabolites deterred predation by spiders (Exp. 3), follow-up experiments 6−9 (*n* = 20 each) tested the effect of each metabolite singly. For each experimental replicate, a synthetic metabolite (40 ng) dissolved in water (50 µl) was applied topically onto a beetle. In all experiments, each spider, beetle and *P. scaber* was tested only once, with the observer blind to the test stimulus.

### 2.5. Statistical analysis

All data were analysed in R using Fisher’s exact tests. We compared data across, rather than within, experiments. Specifically, we compared spider predation on *T. molitor* beetles (Exp. 1) and on chemically well-defended *P. scaber* woodlice (Exp. 2). We compared as well spider predation on water-only-treated beetles (Exp. 4) and on beetles that were topically treated with (i) *P. scaber* gland secretion and water (Exp. 3); (ii) a blend of the four synthetic metabolites **1**, **2**, **9** and **10** (Exp. 5); or (iii) single metabolite **1** (Exp. 6), **2** (Exp. 7), **9** (Exp. 8), or **10** (Exp. 9).

## 3. Results

### 3.1. Identification of defensive metabolites in tegumental gland secretions

**Figure 1.**
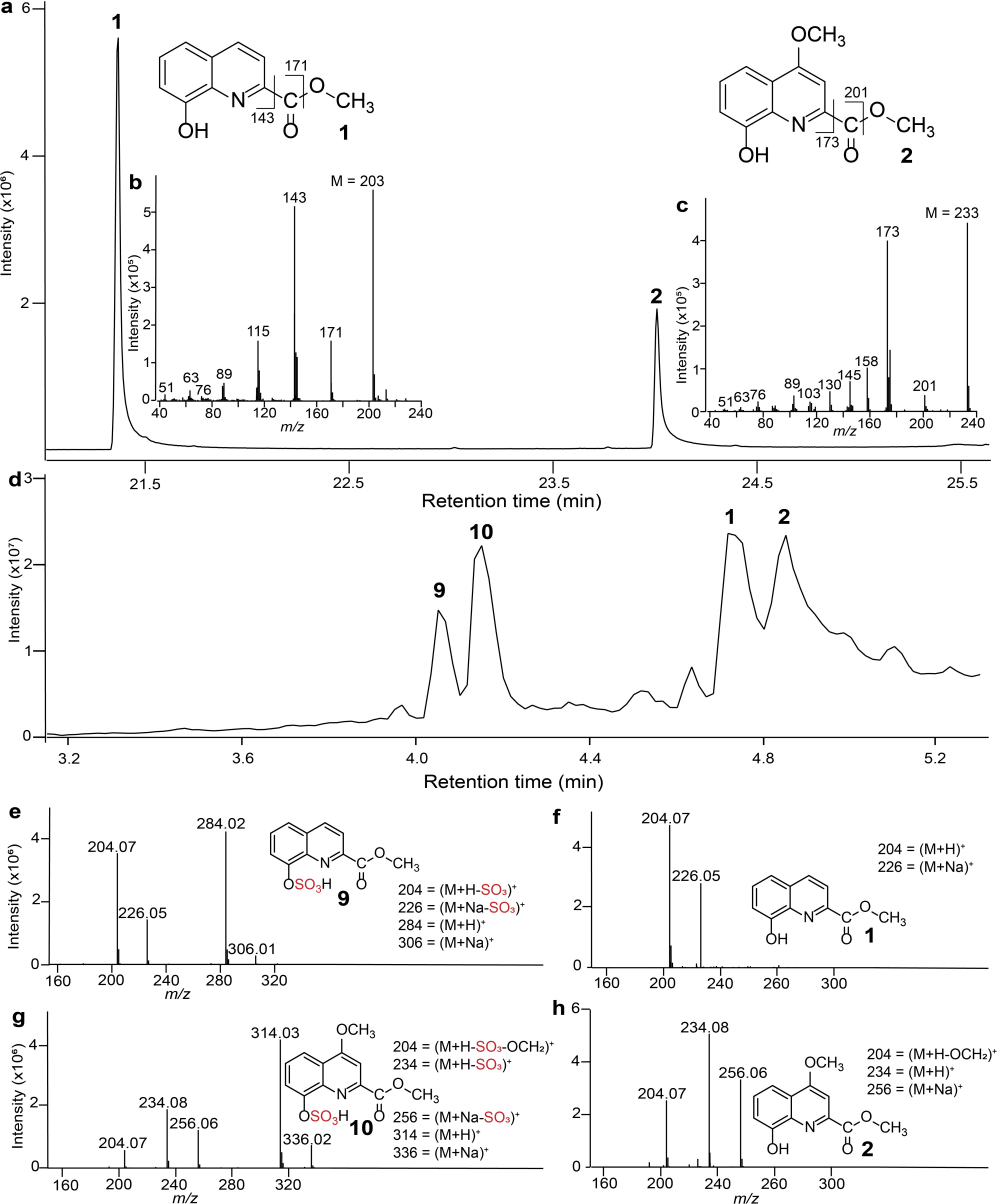
Chromatographic-mass spectrometric analyses of defensive secretions of *Porcellio scaber*. (a) Total ion chromatogram (TIC) of GC-MS. (b, c) Electron ionization spectra of (b) methyl 8-hydroxy-quinoline-2-carboxylate (**1**) and (c) methyl 8-hydroxy-4-methoxy-quinoline-2-carboxylate (**2**). (d) TIC of HPLC-MS. (e−h) Electrospray ionization spectra of (e, f) methyl 8-(sulfooxy)quinoline-2-carboxylate (**9**) and **1,** and of (g, h) methyl 4-methoxy-8-(sulfooxy)quinoline-2-carboxylate (**10**) and **2**.

The total ion chromatogram (TIC; GC-MS) of *P. scaber* tegumental gland secretions revealed two distinct metabolites (**1** and **2** in figure 1a). The molecular ion *m/z* 203 in the mass spectrum of unknown **1** (figure 1b) indicated the presence of a nitrogen-containing compound, probably with aromatic ring structures. This inference was inspired by *m/z* 51, 63 and 76, which are reminiscent of aromatic heterocyclic quinoline. Considering potential biosynthetic pathways (see below), we predicted that unknown **1** had a quinoline rather than an isoquinoline skeleton. The nominal mass of compound **1** (203) exceeded that of quinoline (129) and implied structural elaboration beyond the parent quinoline scaffold. The presence of a carboxylic acid or a methyl ester functionality was supported by *m/z* 143 (203-60), and the presence of a methoxy group by *m/z* 171 (203-32). The tailing peak shape of **1** in the GC-MS TIC (figure 1a) further suggested the presence of a polar hydroxyl group. All data combined led us to hypothesize that **1** is a methyl hydroxyquinoline-carboxylate. To test our hypothesis, we selectively methylated the acid group of 4-hydroxy-quinoline-6-carboxylic acid (**3**), 5-hydroxy-quinoline-3-carboxylic acid (**4**) and 8-hydroxy-quinoline-2-carboxylic acid (**5**) using catalytic sulfuric acid in methanol. The methylation product of **5**—methyl 8-hydroxy-quinolene-2-carboxylate—had retention and mass spectral characteristics identical to unknown **1**. Unknown **2** (figure 1a), with a nominal mass of 233 (figure 1c), was thought to be a homologue of **1** with an additional methoxy functionality. Drawing on the known biosynthesis of **5** via xanthurenic acid (4,8-dihydroxyquinoline-2-carboxylic acid (**6**)) in the kynurenine pathway [31], we proposed two structural candidates for unknown **2**: methyl 8-hydroxy-4-methoxy-quinoline-2-carboxylate (**7**) and methyl 4-hydroxy-8-methoxy-quinoline-2-carboxylate (**8**). Compound **8** did not match unknown **2** but could be used to produce methyl 8-hydroxy-4-methoxy-quinoline-2-carboxylate (figure 1c) (electronic supplementary material), which had retention and mass spectral characteristics consistent with unknown **2**.

**Figure 2.**
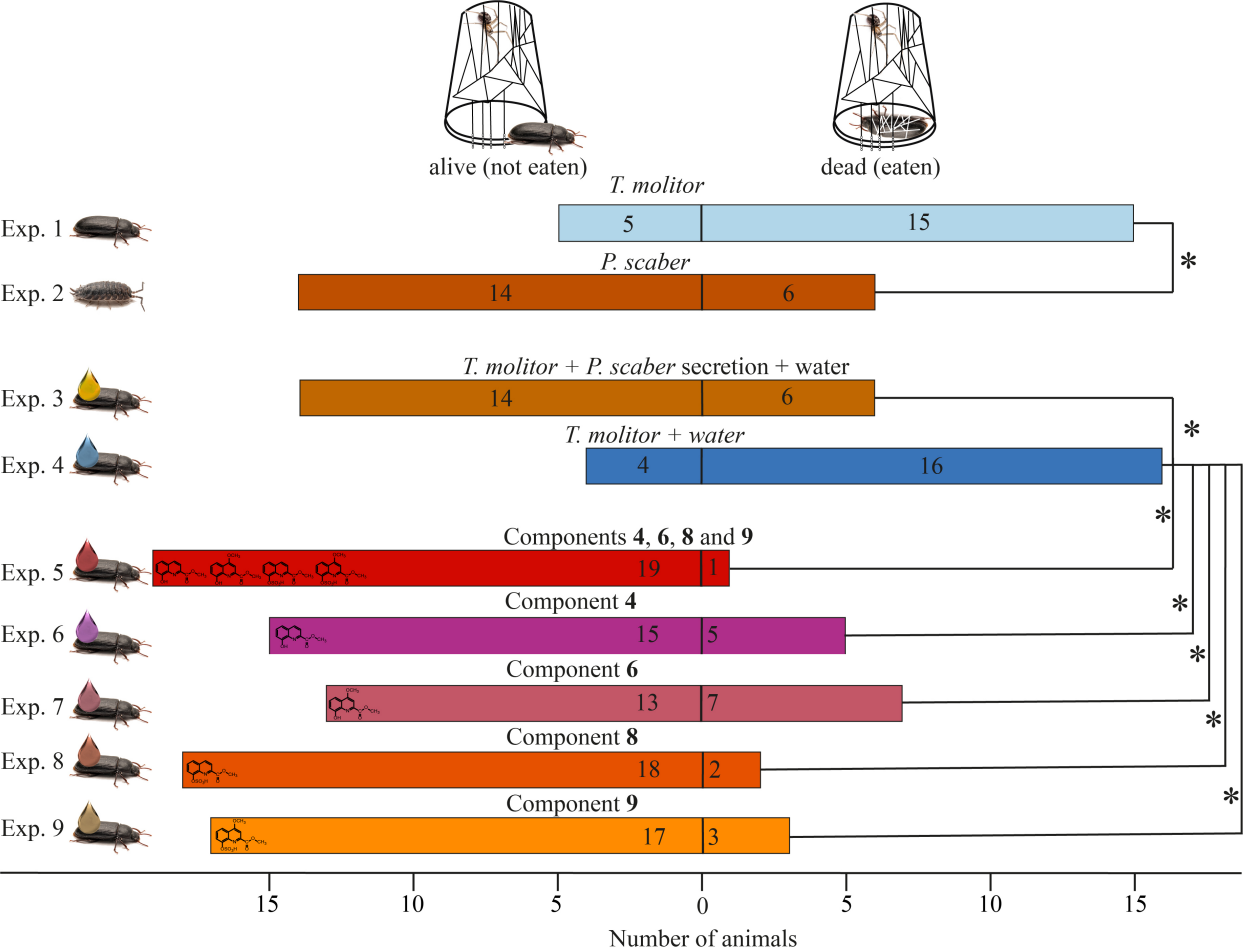
Effects of (i) prey type (chemically well-defended *P*. *scaber* woodlice or chemically less defended *T*. *molitor* beetles) and (ii) prey treatment (topical application of beetles with *P. scaber* gland secretion, synthetic gland metabolites (40 ng) or a water control) on predation by *S*. *grossa* false widow spiders. Bars indicate the number of prey being preyed upon and killed (right of 0), or not (left of 0), by the spiders. Metabolites: **1** = methyl 8-hydroxy-quinoline-2-carboxylate; **2** = methyl 8-hydroxy-4-methoxy-quinoline-2-carboxylate; **9** = methyl 8-(sulfooxy)quinoline-2-carboxylate; **10** = methyl 4-methoxy-8-(sulfooxy)quinoline-2-carboxylate. An asterisk (*) denotes a statistically significant difference (Fisher’s exact tests; *p* < 0.05).

LC-MS analysis of *P. scaber* secretion extract confirmed the presence of **1** and **2**, and revealed unknown **9** and **10** (figure 1d). Positive ESI spectra of **9** (figure 1e) and **1** (figure 1f) suggested structural similarities, based on near identical fragment ions **(9**: *m/z* 204.07 (M+H–SO_3_)^+^; *m/z* 226.05 (M+Na–SO^3^)^+^; **1**: *m/z* 204.07; *m/z* 226.05). The additional fragment ions in the spectrum of **9** *(m/z* 284.02 (M+H)^+^, *m/z* 306.01 (M+Na)^+^), and the nominal mass differential (79.95) between protonated **9** (*m/z* 284.02) and **1** (*m/z* 204.07), suggested the presence of a –SO_3_ group (79.95 Da) in **9**. Similarly, the ESI spectra of **10** (figure 1f) and **2** (figure 1h), revealing near identical fragment ions (**10**: *m/z* 204.07; *m/z* 234.08; *m/z* 256.06; *m/z* 314.03 (M+H)^+^; **2**: *m/z* 204.07; *m/z* 226.05; *m/z* 234.08; *m/z* 256.06) suggested structural similarities between **10** and **2**. The additional fragment ions in the spectrum of **10** *(m/z* 256.06 (M+Na–SO_3_)^+^; *m/z* 314.03 (M+H)^+^; *m/z* 336.02 (M+Na)^+^), and the nominal mass differential (79.95) between protonated **10** (*m/z* 314.03) and **2** (*m/z* 234.08), suggested again the presence of an –SO_3_ group in **10**. Drawing on the biosynthetic kynurenine pathway [31], we predicted the sulfooxy group to be at C-8, resembling 3-hydroxykynurenine-*O*-sulfate produced by bed bugs [32]. Thus, we synthesized both methyl 8-(sulfooxy)quinoline-2-carboxylate and methyl 4-methoxy-8-(sulfooxy)quinoline-2-carboxylate (electronic supplementary material), which had retention and mass spectrometric characteristics consistent with unknown **9** and **10**, respectively. With authentic standards of metabolites **9**, **10**, **1** and **2** in *P. scaber* gland secretion extract at hand, we determined that the tegumental gland secretions of a single *P. scaber* woodlouse contained, on average (1 equivalent), 12 ng of **1**, 18 ng of **2**, 4 ng of **9**, and 6 ng of **10**.

### 3.2. Behavioural results

Prediction 1*—S. grossa* preys more readily on *T. molitor* than on chemically well-defended *P. scaber*—was supported by data in experiments 1−4. Spiders preyed on 15 out of 20 beetles (Exp. 1) but preyed on only 6 out of 20 *P. scaber* (Exp. 2) (*p* = 0.010, Fisher’s exact test, figure 2). Similarly, spiders preyed on only 6 out of 20 beetles treated with *P. scaber* secretions extracted in water (Exp. 3) but preyed on 16 out of 20 beetles treated only with water (Exp. 4) (*p* = 0.004, Fisher’s exact test, figure 2).

Prediction 2*—S. grossa* rejects *T. molitor* rendered chemically well-defended by treatment with synthetic *P. scaber* secretion metabolites—was supported by data in Exp. 5. Spiders preyed on only 1 out of 19 beetles treated with a synthetic blend of **1**, **2**, **9** and **10** (at 1 woodlouse equivalent) but preyed on 16 out of 20 beetles treated only with water (Exp. 4) (*p* < 0.001, Fisher’s exact test, figure 2). Compared with spider predation on water-only-treated beetles (Exp. 4), each synthetic woodlouse metabolite applied onto beetles (at 40 ng) protected beetles from predation (**1** in Exp. 6: 15 (alive) versus 5 (dead), *p* = 0.001; **2** in Exp. 7: 13 versus 7, *p* = 0.009; **9** in Exp. 8: 18 versus 2, *p* < 0.001; **10** in Exp. 9: 17 versus 3, *p* < 0.001, Fisher’s tests, figure 2).

## 4. Discussion

Our data support the hypothesis that the crustacean terrestrial woodlouse *P. scaber* produces defensive metabolites that provide protection against terrestrial predators. We show that the quinoline derivates **1**, **2**, **9** and **10** in the tegumental secretions of *P. scaber* deter predation by a terrestrial spider. With these chemical and behavioural data, there is now unequivocal evidence that the crustacean *P. scaber*, like insects [5,33], is chemically defended against a predatory spider [16]. As each of the four quinoline derivates on its own deterred predation by a spider, the blend of all four derivates appears to be a surefire mechanism that ensures protection against a diverse guild of terrestrial predators.

The presentation of multiple behaviour-modifying metabolites is favoured in predator–prey arms races [34] because predators co-evolving with prey may adapt and overcome prey defences [35]. The predator–prey arms race invokes the evolution of novel defensive traits, resulting in the diversification of chemical armaments. Interestingly, chemical defences are thought to diversify less rapidly than the offensive venoms of predators. Venomous predators exert strong resistance selection in their prey populations by removing vulnerable prey, with venom resistance, in turn, amplifying counter-selection for novel offensive agents [36]. On the other hand, in prey evolving deterrence against predators does not require the death of prey, but only a bite attacking predators will remember [34,37]. Predators are often generalists and could switch to other prey while maintaining some offensive potency despite evolving prey defences [34,37]. Costly offensive or defensive metabolites that do not accrue substantial fitness benefits are expected to be purged over evolutionary time [36]. Considering both the functional need for varied offensive and defensive metabolites and their production costs, one can expect greater diversity in offensive than in defensive metabolites [34].

Predators with particularly effective co-adaptations to circumvent protective traits of their prey are favoured to specialize on that prey. Such specialized predators of terrestrial isopods are woodlouse spiders in the genus *Dysdera* [38]. *Dysdera* spiders have long chelicera mouthparts that can pierce the hard armour of isopods [39] and help minimize contact with the isopods’ tegumental gland secretions. The heavy (molecular weight greater than 200 Da) and polar metabolites **1**, **2**, **9** and **10** embedded in the proteinaceous [5,20] gland secretions of *P. scaber* are probably sensed by tip-pore sensilla at the mouth opening of spiders [40], providing gustatory information on the prey prior to ingestion [41]. Likewise, ants perceive prey chemicals based on receptors on their antennae [20,42]. By secreting distasteful metabolites from their tegumental glands, isopods inform predators about their distastefulness and thereby deter predation. Some predators, however, co-adapted traits that circumvent these prey defences [38]. Studying the chemical defences of diverse terrestrial isopods against their specialist *Dysdera* predators would provide insight into evolutionary co-adaptations in predator–prey arms races [16,38] and would help us understand the selective pressures that have shaped the diversity of defensive mechanisms in isopods. This knowledge would also shed light on the co-evolutionary dynamics—and their effects on biodiversity—between predator and prey that were at play when crustaceans colonized land [43].

With reports that tegumental gland secretions of terrestrial Oniscidea isopods, including those of *P. scaber*, deter predators ([19,20], this study), it would be intriguing to investigate the chemical defences of aquatic isopods against aquatic predators, and to compare their metabolites with those identified in this study. To date, it is only known that juveniles of the benthic isopod *Glyptonotus antarcticus* are chemically defended because extracts of juveniles were unpalatable to their sea star predator *Odontaster validus* [44]. Functions of the *P. scaber* tegumental gland metabolites other than predator deterrents, such as microbial defence or desiccation resistance, are not likely because these metabolites are contained in the tegumental glands and are secreted only upon a predatory threat [18]. Moreover, upon secretion from the glands, the metabolites quickly coagulate [5] and cannot spread over the body surface, thus not functioning in microbial defence or desiccation resistance.

There is limited knowledge about the metabolites we identified in *P. scaber* secretions. Compound **2** was not previously known, nor were the sulfooxy quinolines **9** and **10**. Reports that compound **1** protects the dytiscid water beetle *Ilybius fenestratus* from frog predation [45] support the concept of convergent evolution, because water beetles secondarily transitioned from terrestrial to aquatic habitats, whereas isopods transitioned from water to land. However, without phylogenetic analysis, horizontal gene transfer or shared ancestry as alternative interpretations cannot be dismissed. The corresponding acid of **1**, 8-hydroxy-4-methoxy-quinoline-2-carboxylic acid (**5**), is produced by larvae of the noctuid moth *Spodoptera littoralis* and believed to serve as an iron-chelating agent, whereas a potential defensive role was not explicitly tested [31,46]. This acid **5**—previously termed ‘quinolobactin’—functions as a siderophore for *Pseudomonas* bacteria, facilitating iron acquisition [47]. Larvae of *S. littoralis* biosynthesize **5** from tryptophan via kynurenine and 3-hydroxykynurenine [31]. As the amount of tryptophan in the diet of these moth larvae determined the amount of **5** they produced [31], it is conceivable that the defensive secretions of *P. scaber* require sufficient tryptophan uptake. In carnivorous cone snails, dietary intake is positively correlated with their venom complexity [48]. Whether *P. scaber* also utilizes dietary precursors for the biosynthesis of its defensive quinoline metabolites remains to be investigated. Locating the biosynthesis site of these quinolines could shed light on the evolution of this defensive trait.

Quinoline derivates are rare natural products but occur in a wide range of taxa including bacteria, plants and animals [49]. The phasmid insect *Oreophoetes peruana* biosynthesizes quinoline from *l*-tryptophan and sequesters quinoline as a predator repellent [50,51]. Quinoline derivates also serve as chemical defences in a lycid beetle [52], a coccinellid ladybird beetle [53], a *Solenopsis* ant and the mantelline frog *Mantella betsileo* [54]. Scolopenolines represent a diverse set of quinoline derivates produced by the centipede *Scolopendra subspinipes mutilans* [55]. Scolopenoline B is a sulfooxy-containing heterodimer comprising quinoline and tyramine moieties [55]. With a sulfooxy group at C-8, scolopenoline B bears resemblance to metabolites **9** and **10** of *P. scaber*.

## 5. Conclusion

We have identified four deterrent metabolites (three previously unknown) in the defensive secretion of *P. scaber,* supporting the hypothesis that crustaceans produce defensive metabolites that provide protection against terrestrial predators. Our data further demonstrate that, like many insects, some terrestrial crustaceans are chemically defended against predators.

## Data Availability

All data and statistical code are provided in the manuscript and in supplementary material which is available online [56].
